# Prudent electrochemical pretreatment to promote the OER by catalytically inert “Iron incorporated metallic Ni nanowires” synthesized *via* the “non-classical” growth mechanism†

**DOI:** 10.1039/d0na00073f

**Published:** 2020-03-16

**Authors:** Athma E. Praveen, Sagar Ganguli, Venkataramanan Mahalingam

**Affiliations:** Department of Chemical Sciences, Indian Institute of Science Education and Research Kolkata Mohanpur Nadia West Bengal India mvenkataramanan@yahoo.com

## Abstract

This study provides new insight towards the non-classical “amorphous to crystalline” growth mechanism for metal nanowire synthesis and reports an electrochemical strategy to activate inactive materials into efficient electrocatalysts for the OER. Despite considerable research on transition metal oxides/hydroxides, especially NiFe based hydroxides as OER electrocatalysts, poor conductivity of these materials plagues their catalytic efficiency. In contrast, lack of catalytic centers hinders the OER performance of conductive metals. Herein, we devised a suitable precondition strategy to transform only the surface of conductive metallic Ni nanowires into active catalytic centers. The resulting material with intimate contact between the electrically conductive core and electrocatalytically active surface showed promising “specific” and “geometric” electrocatalytic activity towards the alkaline OER at different pH. Upon iron incorporation, the Fe centers incorporated at the surface as well as in the bulk of the nanowires were found to further boost the OER activity of these materials. A one-pot strategy was adopted to produce iron free/incorporated Ni nanowires covered with nano-spikes. Growth analysis revealed a unique “non-classical amorphous-to-crystalline transformation” to be responsible for the formation of metallic nanowires.

## Abstract 2

This study provides new insight towards the non-classical “amorphous to crystalline” growth mechanism for metal nanowire synthesis and reports an electrochemical strategy to activate inactive materials into efficient electrocatalysts for the OER.

## Graphical abstract

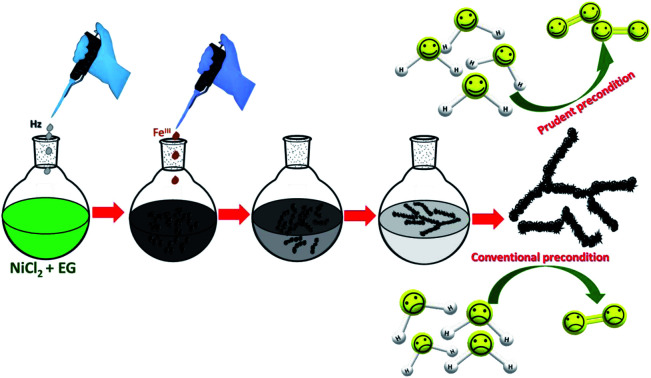

## Introduction

Increase in energy demand with the advancement of civilization and the environmental ramifications associated with fossil fuel usage have stimulated the research on energy generation from renewable energy sources in recent decades. However, due to the intermittent nature of renewable energy sources, storage of surplus energy is a necessity. In this context, storage of energy in the form of chemical energy, especially in hydrogen (H_2_), is receiving increased attention due to its high energy density and environmentally benign nature. Water electrolysis, ideally driven by a renewable source (solar, wind or tidal sources), is one of the most efficient and sustainable ways to produce hydrogen.^1,2^ Water electrolysis involves two half-reactions, the hydrogen evolution reaction (HER) at the cathode and the oxygen evolution reaction (OER) at the anode. Among them, the reaction dynamics of the OER are greatly constrained due to high activation energy barriers, and electrocatalysts are generally employed to conduct the reaction at a reasonable rate. At present, even in the presence of state-of-the-art noble metal catalysts (such as RuO_2_ and IrO_2_), a substantial overpotential is required to drive the oxidation of water.^3,4^ The high-cost and scarcity of noble metal catalysts demand for the development of low-cost, abundant, and transition metal based efficient OER catalysts. Several first-row transition metal oxides and hydroxides have been reported to show promising electrocatalytic performance, especially late transition metal hydroxides such as Co(OH)_2_-TCNQ,^5^ NiCo_2_O_4_@Ni–Co–Ci,^6^*etc.* Among transition metal oxides and hydroxides, Ni-based oxides and hydroxides show superior OER activity in the alkaline medium.^7^ In particular, Fe incorporated Ni-based oxides and hydroxides such as NiFe(OH)_*x*_,^8^ NiFe/NF,^9^ NiFe-LDH@NiFe–Bi/CC,^10^ Fe–NiCr_2_O_4_/NF,^11^ NiFeO_*x*_,^12^*etc.* have received lot of attention due to their stellar performance in the OER. Despite significant advancements in the field of transition metal hydroxide based electrocatalysts, their low electrical conductivity severely limits their electrocatalytic performance. In contrast, metallic nanoparticles/nanowires are known to be highly conductive but suffer from poor electrocatalytic activity. This has enthused researchers to develop composites of metal hydroxides with metallic nanostructures.^13,14^ However, the drawback of conventional methods for composite synthesis arises from the poor contact between the different components. This demands for the development of an approach that will produce materials with superior contact between the electrically conductive and electrocatalytically active components.^15^

In this work, we have addressed this concern by devising a strategy to electrochemically activate iron free/incorporated metallic nickel nanowires synthesized at low temperature. Briefly, a simple wet-chemical method was employed to synthesize nickel nanowires and iron [Fe(iii)] incorporated nickel nanowires without the assistance of any templates and external magnetic field.^16^ Interestingly, sharp nano-spikes were found to uniformly form on the surface of these nanowires. A detailed analysis of the growth mechanism was performed that reveals that the formation proceeds through a non-classical pathway of nanoparticle attachment followed by a unique “amorphous to crystalline” transformation of the as-synthesized materials. The initial OER activity of the materials was found to be poor. The underlying reason was probed and the problem was found to primarily arise from the incompatibility of the widely used precondition step in generating electrocatalytically active sites. The precondition step was modified by prudent choice of potentials and electrochemical techniques and this resulted in significant improvement of the electrocatalytic OER activity. Rigorous post-catalytic analysis was performed and it revealed that the modified precondition step generates a thin-layer of nickel hydroxide on the surface of nickel nanowires. While the nickel hydroxide layer at the surface efficiently electrocatalyzes the OER, the metallic nickel core provides the much-needed electrical conductivity. The iron incorporated nickel hydroxide was found to show significantly better activity from both “intrinsic” and “geometric” perspectives. Controlled studies indicated that along with the Fe(iii) ions present at the surface of nickel nanowires, those incorporated in the bulk also participate in the promotion of the electrocatalytic OER activity.

## Experimental section

### Synthesis of nickel iron nanowires (NiFe NWs)

An aqueous solution consisting of 5 mM NiCl_2_ was added to 15 mL of ethylene glycol in a round bottom flask. The mixture was then heated in an oil bath at 110 °C. About 1 mL of hydrazine hydrate solution was rapidly added into the hot solution without any stirring. Immediately after the addition, the whole solution turned black in color. After ∼1 min, aqueous solutions containing FeCl_3_ in different amounts were added to achieve the final iron concentrations of 2.5 mM, 5 mM, and 10 mM in the resulting reaction mixtures. Subsequently, black products were found floating over the solution. These were then collected and washed several times with distilled water and ethanol to remove excess unreacted reactants and solvent. For ease of understanding, the as-synthesized materials have been denoted based on the molar ratio of different metal precursors used during synthesis. For example, the materials synthesized from aqueous solutions consisting both NiCl_2_ and FeCl_3_ at 5 mM concentration have been termed NiFe (1 : 1).

### Synthesis of nickel NWs (Ni NWs)

For a comparative account, Ni NWs were synthesized following the same protocol for NiFe NWs except for the addition of FeCl_3_. The resulting black product was washed several times with distilled water and ethanol to remove excess hydrazine and solvent.

## Results and discussion

### Structural characterization

#### Phase analysis

**Fig. 1:**
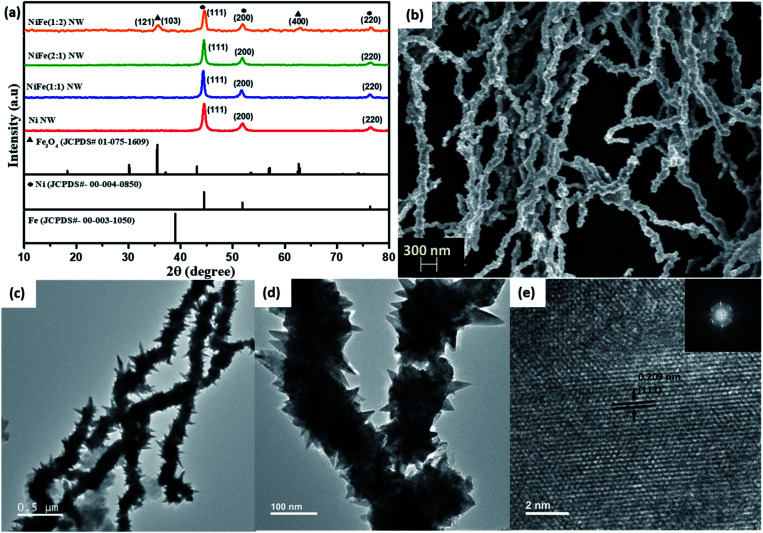
(a) PXRD patterns of the Ni and NiFe materials. (b) SEM and (c–e) TEM images of the as-prepared NiFe (1 : 1) nanowires.

To obtain information regarding the structure of the as-synthesized products, PXRD patterns of the materials were collected (Fig. 1a). The PXRD pattern of Ni was found to perfectly match with the standard pattern of metallic nickel (ICDD #04-0850).

Interestingly, in the diffraction patterns of NiFe (2 : 1) and NiFe (1 : 1), no characteristic peaks from either iron or iron oxides were observed. Instead, peaks at 2*θ* = 44°, 51° and 76° values corresponding to the (111), (200), and (220) planes of metallic nickel, respectively, were found to be present. The absence of any other peaks from oxides of nickel and iron implies that metallic nickel is the primary constituent in these products. In contrast, additional peaks corresponding to Fe_3_O_4_ (ICDD #75-1609) along with those of metallic nickel were observed in the diffraction pattern for NiFe (1 : 2), thereby suggesting that using an excess amount of iron compared to nickel leads to the formation of iron oxides.

#### Elemental analysis

To ascertain whether any iron is incorporated in NiFe (2 : 1) and NiFe (1 : 1) and to know the exact elemental compositions, ICP-AES analysis of all three NiFe materials was carried out. The atomic ratio of nickel and iron was found to be 11.62 : 1 for NiFe (1 : 1), 1.01 : 1 for NiFe (1 : 2) and 473.8 : 1 for NiFe (2 : 1) (shown in Table 1). This confirms the presence of iron in all three materials. The higher proportion of nickel over iron in NiFe (1 : 1) and NiFe (2 : 1) along with an almost identical proportion in NiFe (1 : 2) indicates that under the experimental conditions, the formation of a solid product from nickel is more favourable compared to that from iron. The colour of the supernatant after synthesis further supports this observation. As shown in Fig. S1a,† the initially green reaction solution turned completely transparent after product formation in the case of Ni NWs thereby indicating that majority of the nickel ions have been consumed during the reaction. In contrast, the colour of the supernatant after the reaction for all three NiFe nanowires was deep brown, which Fe^3+^ ions are known to impart in aqueous solutions (Fig. S1b†).

**Table 1:** ICP-AES analysis data and the Ni : Fe element ratio in NiFe NWs

Sample	Ni (ppm)	Fe (ppm)	Ni : Fe ratio
NiFe (1 : 1)	63.522	4.839	11.62 : 1
NiFe (1 : 2)	25.959	24.343	1.01 : 1
NiFe (2 : 1)	50.083	0.106	473.8 : 1

#### Morphology analysis and elemental mapping

The morphology of the as-prepared samples was investigated using electron microscopy. In the SEM analysis of Ni, NiFe (2 : 1) and NiFe (1 : 1), formation of long wires with a non-uniform surface was observed (Fig. 1b and S1c–e†). TEM analysis also reveals a similar observation for all the materials. The nanowires were found to have a length of ∼5–6 μm and diameter of ∼120 nm (Fig. 1c, d and S2†). Interestingly, in the TEM images for all of these materials, sharp and thin spike-like structures protruding from the surface of the wires were observed. Moreover, for NiFe (1 : 2), small sized irregular spheres were also found to be present in the TEM images along with the wires (Fig. S2d and f†). The HR-TEM analysis on the spheres shows that the lattice planes correspond to a *d* spacing of 0.254 nm (Fig. S2f†). Upon comparing with the PXRD pattern of this sample, this can be assigned to the (103) plane of Fe_3_O_4_. Therefore, we believe that while the nanowires represent Fe-incorporated Ni, the spheres belong to Fe_3_O_4_ that has formed due to the usage of excess amounts of FeCl_3_.

**Fig. 2:**
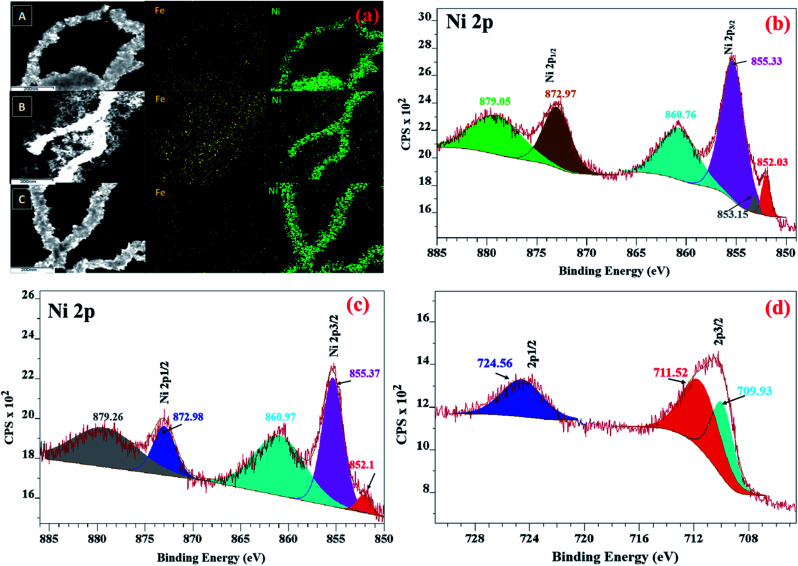
(a) Elemental mapping of (A) NiFe (1 : 1), (B) NiFe (1 : 2) and (C) NiFe (2 : 1) NWs. XPS spectra of the (b) Ni 2p of Ni NWs, (c) Ni 2p and (d) Fe 2p of NiFe NWs.

To understand the spatial distribution of elements, elemental mapping of NiFe nanowires (shown in Fig. 2a) was performed for the as-synthesized materials. In all cases, the nanowires were found to be primarily composed of nickel and with uniform distribution of iron throughout them.

Thus, the morphological and elemental mapping data complement the inferences drawn earlier from PXRD analysis that relative proportion of nickel and iron precursors plays a decisive role in the phase of the final products.

#### Growth mechanism analysis

To understand the evolution of the growth of nanowires with protruding nano-spikes, time-dependent microscopy analysis was performed (Fig. S3†). For this purpose, aliquots were drawn out from the reaction mixture at different intervals during the synthesis and subjected to instant cooling to quench further growth. They were subsequently analyzed through microscopy analysis. Initially, *i.e.*, just after the addition of FeCl_3_, the formation of small (∼30 nm) particles was observed. The absence of any lattice planes in HR-TEM analysis and any sharp points in the FFT patterns clearly suggest that these particles are amorphous in nature. TEM images for aliquots drawn after 60 seconds of reaction after addition of FeCl_3_ reveal that the initially formed small particles undergo coalescence to form larger spheroids (∼120 nm) with irregular surfaces to decrease their surface energy. These larger spheroids then assembled along a particular direction into necklace-like chains due to the anisotropic magnetic forces.^17–20^ The fact that the average diameter of the wires remained similar to that of the spheroids, while the length increased with increase in reaction time validates this point. As highlighted in Fig. S3B(g),† further coalescence of particles on the already formed large spheroids was found to produce small elongations on the surface of spheroids. We believe that they transform into sharp nano-spikes with increase in the reaction time. Interestingly, the materials were found to become crystalline with the passage of reaction time. This is evident from the observation of lattice fringes in HR-TEM images as well as bright and sharp spots in the corresponding FFT patterns. For example, the HR-TEM analysis of NiFe (1 : 1) revealed the presence of lattice fringes corresponding to a *d*-spacing of 0.209 nm, which can be ascribed to the (111) plane of nickel (shown in Fig. 1e). This suggests that with increase in reaction time, an “amorphous to crystalline” transition is taking place during the growth of the metallic nanowires. Francis *et al.* have recently reported the observation of a similar phenomenon during Bi nanowire formation. They concluded that once the amorphous precursor nanoparticles come into contact, a rapid atomic rearrangement takes place to obtain a similar orientation across the materials and this leads to the formation of a single crystalline structure with the passage of reaction time.^21^ In contrast to the traditionally accepted “classical homogeneous nucleation and growth mechanism” which suggests that a crystalline nucleus is first generated, the growth of NiFe NWs was observed to proceed through initial formation of an amorphous dense “pre-nucleus” that subsequently reorders into a thermodynamically stable crystalline nucleus with the passage of reaction time. Such “amorphous to crystalline” transition of nanomaterials is commonly termed the “non-classical growth mechanism”.^22–25^

#### XPS analysis

To understand the surface layer composition, XPS analysis was performed. For Ni NWs (Fig. 2b), a small peak was observed in the narrow scan of Ni 2p at 852.03 eV that represents the metallic (0) state of nickel.^26^ In addition, peaks at 853.15 eV (2p_3/2_), 855.3 eV (2p_3/2_), and 872.97 eV (2p_1/2_), and satellites peaks at 860.76 eV (2p_3/2_) and 879.05 eV (2p_1/2_) were observed, which suggests the presence of Ni(ii) according to literature reports.^27–30^ Thus, though the material is primarily composed of Ni(0) as suggested by PXRD, some amount of Ni(ii) is present at the surface of the as-synthesized material. Additionally, the survey scan reveals the presence of oxygen in the material. In the narrow scan of the O 1s spectrum, a peak was observed at 529.6 eV and has been reported to arise from lattice oxygen (O^2−^) (Fig. S4†).^31^ This suggests that the observed Ni(ii) centers are probably arising due to the formation of some nickel oxide phases at the surface of the Ni NWs and not from any adsorbed Ni(ii) species. In the XPS spectrum of NiFe NWs, the characteristic peak of metallic nickel at 852.11 eV was observed (Fig. 2c). Shifting of Ni peaks to higher energy (∼0.3 eV) upon incorporation of iron confirms the presence of an interaction between nickel and iron centers. For NiFe also, the peaks at 855.67 eV and satellite at 860.97 eV in the Ni 2p narrow scan indicate the presence of Ni(ii) species.^32^ The Fe 2p XPS spectrum exhibited Fe 2p_3/2_ peaks at 709.93 eV and 711.5 eV belonging to Fe^2+^ and Fe^3+^, respectively (shown in Fig. 2d). In addition, the characteristic Fe 2p_1/2_ peak of Fe^3+^ at 724.56 eV was observed.^33^ For NiFe NWs, the presence of oxygen at the surface was detected from the survey scan of XPS and the narrow scan of O 1s reveals that it results from lattice oxygen. Thus, some nickel oxide formation at the surface takes place in this case as well. These observations suggest that though the reaction conditions were able to reduce most of the Ni(ii) to metallic Ni(0), it was not sufficient enough to reduce Fe(iii) to the corresponding metallic Fe(0). Instead, some of the Fe(iii) actually got reduced to Fe(ii) under the reaction conditions.

#### Raman analysis

To identify the actual species formed on the surface of the as-synthesized materials, Raman spectra of Ni NWs and NiFe (1 : 1) were collected. For Ni NWs, two peaks at 540 cm^−1^ and 1075 cm^−1^ were observed (shown in Fig. S5†). Chaki *et al.* have reported that these peaks are characteristic of one phonon (1P) longitudinal optical (LO) and one photon (1P) transverse optical (TO) modes of NiO.^34^ Upon Fe incorporation, both of these peaks were found to be slightly right-shifted (570 and 1095 cm^−1^).

This may be ascribed to the size-induced phonon confinement effect. Recently, Pan *et al.* have also observed a similar phenomenon and attributed it to an interaction between NiO and Fe resulting in decrease in the coupling interaction of Ni–O oscillation.^35^ Therefore, both XPS and Raman analyses indicate the formation of a thin NiO layer on the surface of metallic Ni/NiFe nanowires. They further reveal strong interaction between the nickel and iron species present in the NiFe materials.

### Electrocatalytic oxygen evolution

#### Activity comparison from the geometric perspective and the role of precondition

The OER performance of the as-synthesized NWs was measured in a three-electrode cell configuration using 1 M KOH, as discussed in the electrochemical measurement section (ESI†). Current density from the backward linear sweep voltammetry (LSV) curves was preferred to that from forward LSV scans to evaluate the electrocatalytic activity as the former is known to be devoid of any contribution from capacitive or metal redox currents and truly represents the electrocatalytic activity of materials.^36,37^ As shown in Fig. S6A,† the OER activity of NiFe NWs from the geometric perspective was found to be better compared to that of only Ni NWs as the current density of the former at any given potential was higher than that of the latter. However, compared to the already available reports on nickel and nickel–iron based materials, the OER activity of the synthesized products fared poorly even after subjecting the electrodes to high overpotentials. This may be due to lower density of catalytically active sites in the as-synthesized materials. According to literature reports, metal hydroxides, especially iron-incorporated nickel(ii) hydroxide, are known to be one of the best transition metal based electrocatalysts for the OER and the higher oxidation states of nickel, such as Ni(iii) and Ni(iv), are the active centers for electrocatalysis for these materials. For nickel(ii) hydroxide, the widely followed step of precondition by CV in the potential window of 1 to 1.7 V *vs.* RHE is enough for catalyst activation as the standard reduction potentials of both Ni(iii)/Ni(ii) and Ni(iv)/Ni(iii) fall within that potential range. However, structural and elemental characterization experiments of the as-synthesized materials have suggested that majority of nickel centers are in the (0) oxidation state. Thus, employing a similar precondition step by CV would not be able to produce the desired active sites for electrocatalysis to a sufficient extent and alternative routes must be explored to increase the electrocatalytic activity by generating a larger number of electrocatalytically active oxidation states of metal on the metal nanowires. According to the literature, the standard reduction potentials for Ni^II^/Ni (*E*^0^_Ni(ii)/Ni_) and Fe^III^/Fe (*E*^0^_Fe(iii)/Fe_) are (−0.23) V and (−0.04) V *vs.* RHE, respectively. Therefore, the fabricated electrodes were first subjected to chronoamperometry at 0.3 V *vs.* RHE for different time intervals followed by 20 cycles of CV in 1 M KOH in the potential range of 1 to 1.7 V *vs.* RHE. This particular potential was chosen because while it would be able to oxidize some of the Ni to Ni(ii), the oxidation states of iron centers will remain unaffected. Due to the highly alkaline environment, the generated Ni(ii) centers will readily transform into the corresponding hydroxide. The subsequent treatment by CV will then be able to generate the higher oxidation states of nickel leading to higher OER electrocatalytic activity. This strategy was found to be effective as significant improvement of geometric current density, which in turn represents improved electrocatalytic activity, was observed after the activation process. To find the optimum activation, *i.e.*, the optimum concentration of nickel hydroxide on the surface of metallic nickel, chronoamperometry was carried out for different times and the activity was found to reach its maximum after conducting it for 60 seconds (shown in Fig. S6B†). We believe that the lowering of activity for materials subjected to chronoamperometry for times longer than 60 seconds is primarily due to the non-conductive nature of the nickel hydroxide layer, the concentration of which is proportional to the precondition duration.

**Fig. 3:**
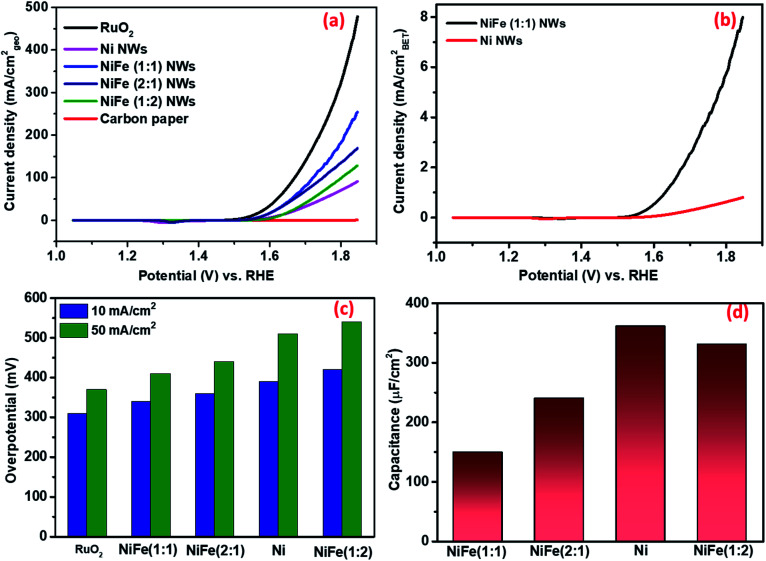
(a) CV curves for RuO_2_, Ni, NiFe (1 : 1, 1 : 2, and 2 : 1), NWs and bare carbon paper in 1 M KOH, normalized by the geometric surface area of electrodes. (b) CV curves for Ni and NiFe (1 : 1) NWs in 1 M KOH, normalized by the BET surface area of samples. (c) Overpotential required for different electrocatalysts to reach current densities of 10 and 50 mA cm_geo_^−2^. (d) The *C*_dl_ calculations of Ni and NiFe (1 : 1, 1 : 2, and 2 : 1) NWs reflecting the relative ECSA of different materials.

As shown in Fig. 3a and c, the overpotential requirements to attain the benchmark current density of 10 mA cm_geo_^−2^ for activated NiFe (1 : 1) and (2 : 1) were considerably lower compared to those for activated Ni. In contrast, the overpotential requirement of NiFe (1 : 2) to reach 10 mA cm_geo_^−2^ was found to be higher compared to that of even Ni. These observations clearly suggest that though incorporation of iron in nickel leads to an improvement in electrocatalytic OER activity, iron itself may not be the active catalytic center as the OER activity significantly decreased for the material NiFe (1 : 2) consisting of separately formed Fe_3_O_4_. The activity of these materials was then compared with that of RuO_2_, which is known to be the state of the art electrocatalyst for the OER and is considered as a global benchmark. While RuO_2_/CP required an overpotential of 310 mV to produce 10 mA cm_geo_^−2^, NiFe (1 : 1) nanowires that fared best in terms of activity among all the synthesized materials needed an overpotential of 330 mV for a similar activity. This suggests that NiFe (1 : 1) may be considered to possess high electrocatalytic activity towards the OER. The overpotential value of NiFe (1 : 1) was compared with literature reports by considering the overpotential requirement for 10 mA cm_geo_^−2^ in the alkaline medium (shown in Table S1†). We would like to caution readers at this juncture that many of the reports have determined the performance of their materials from a forward scan that is convoluted from capacitance and redox currents. As this results in inflated activity, a better overpotential value compared to NiFe (1 : 1) does not truly reflect better activity.

To further understand whether NiFe (1 : 1) is able to perform efficiently at various pH, electrochemical measurements were performed in 0.1 M and 30 wt% KOH solutions. As shown in Fig. S7A,† it required 260 mV, 370 and 530 to reach 20 mA cm_geo_^−2^ in 30 wt% KOH, 1 M KOH and 0.1 M KOH, respectively. Such improvement of electrocatalytic activity upon increased KOH concentration, particularly in 30 wt% KOH, indicates the suitability of the material in high alkaline environments that is employed in commercial electrolyzers.^38^

Furthermore, to understand whether the electrocatalytic activity of NiFe (1 : 1) is higher than that of Ni under neutral conditions, electrochemical measurements were carried out in 1 M phosphate buffer solution (PBS) after activating the catalysts in 1 M KOH through the precondition strategy. As shown in Fig. S7B,† the overpotential requirements to attain 10 mA cm_geo_^−2^ current density for NiFe (1 : 1) and Ni were 620 mV and 680 mV, respectively. Thus, the OER electrocatalytic activity of NiFe (1 : 1) was superior to that of Ni even under neutral conditions.

#### Role of iron incorporation behind activity enhancement

To understand the factors that lead to enhanced electrocatalytic OER activity upon iron incorporation in nickel, further experiments were carried out.

##### (i) Electrochemically active surface area (ECSA) measurement

In general, the ECSA is considered as one of the defining factors behind the electrocatalytic activity of any material. Several recent reports have suggested that the electrocatalytic activity of materials increases with the increase in ECSA. However, due to the limitations of all of the existing methods, accurate determination of ECSA is extremely challenging. Surendranath *et al.* have reported that the double layer capacitance (*C*_dl_) value of a material measured in non-aqueous electrolytes, such as KPF_6_/acetonitrile, is proportional to the ECSA for transition metal based compounds (Fig. S8 and S9†).^39^ This approach was first adopted to estimate the relative ECSA of different as-synthesized materials. Interestingly, despite its poor activity, the *C*_dl_ of Ni (361 nF cm^−2^) was found to be the highest among all the materials as NiFe (2 : 1), NiFe (1 : 2) and NiFe (1 : 1) possess *C*_dl_ values of 331, 240, and 150 nF cm^−2^, respectively (as shown in Fig. 3d). We believe that the increase of OER activity with a decrease in ECSA, in particular for NiFe (1 : 1), clearly suggests that the enhanced activity does not arise due to the presence of a higher number of active sites. Rather, it may be ascribed to the presence of more potent catalytic centers that are able to catalyze the reactions with enhanced efficiency.

##### (ii) Intrinsic/specific activity determination

In recent times, several research groups have pointed out that widely used parameters, such as the current density per unit geometric area of the electrode and comparison based on the overpotential requirement to attain the benchmark current density of 10 mA cm_geo_^−2^, do not represent the intrinsic electrocatalytic activity of materials as they are influenced by loading of catalyst materials as well as other external factors.^40^ As a result, several other parameters have been proposed to compare materials on the basis of their intrinsic activity. Among them, the specific activity, *i.e.*, current density normalized by the BET surface area of materials has been found to have a reasonable reflection of ECSA normalized specific activity for dropcast electrodes. Recently, the specific activity of oxide particles determined from BET surface area was found to agree with that measured for well-defined epitaxial oxide thin-film surfaces, which is considered as a superior method for specific activity estimation.^41^

**Fig. 4:**
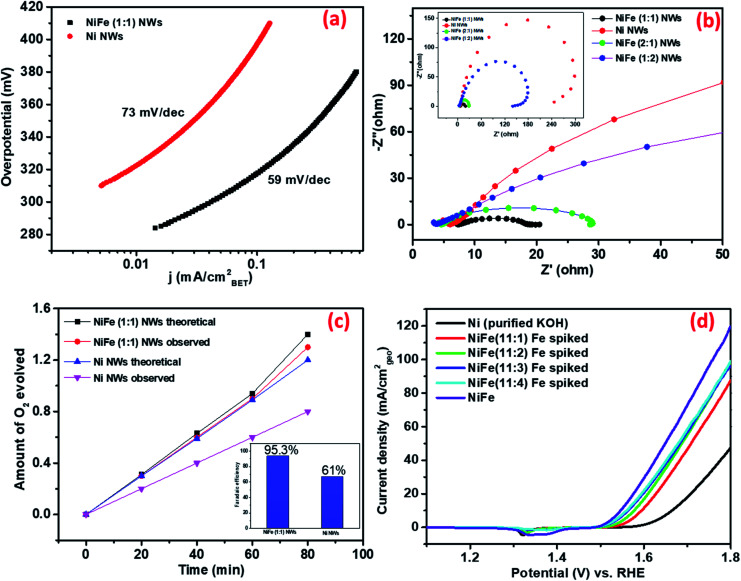
(a) Tafel plots of NiFe (1 : 1) and Ni NWs. (b) The EIS curves of Ni and NiFe (1 : 1, 1 : 2, and 2 : 1) NWs measured at 1.55 V *vs.* RHE. (c) The amount of theoretically calculated and experimentally measured oxygen over time using the Ni and NiFe as electrocatalysts at 1.6 V *vs.* RHE for 20 min (inset shows the faradaic efficiency). (d) CV curves of Ni and NiFe in Fe spiked KOH and purified KOH.

Therefore, the obtained current of only Ni and NiFe (1 : 1) was normalized by BET surface area. As shown in Fig. 3b, the enhancement of activity in NiFe (1 : 1) compared to Ni at any potential was found to be higher compared to that reflected from geometric current density. This further validates the earlier inference that the higher activity for NiFe materials actually arises due to the presence of more potent active sites. The Tafel slopes obtained from BET normalized current density further support this observation (shown in Fig. 4a). The calculated Tafel slope for NiFe (1 : 1) NWs was 59 mV dec^−1^ whereas the corresponding value for Ni NWs was much higher (73 mV dec^−1^) thereby suggesting faster kinetics in the iron incorporated material due to the presence of sites with higher catalytic potency.

##### (iii) Bulk *vs.* surface atoms

In the recent past, several research groups have investigated the effect of iron incorporation on the electrocatalytic activity of nickel oxides/hydroxides in great detail. Boettcher *et al.* have recently reported that the improved activity primarily arises from the iron centers incorporated at the surface.^42^ To confirm whether the enhancement in the electrochemical activity of the as-synthesized materials arises from iron centers present at the surface of the Ni NWs or from those present in the bulk, OER measurements of the Ni NW catalyst were conducted by directly exposing it to purified KOH electrolyte (details of preparation in the ESI†) spiked with different concentrations of Fe(iii) ions. Polarization curves in Fig. 4d show that the OER activity of Ni NWs significantly improved even in the most diluted iron spiked KOH. During spiking experiments, the Fe(iii) ions generally get incorporated at the surface of Ni NWs. Upon comparison, the OER activity of NiFe (1 : 1) was found to be better compared to that of Fe spiked Ni NWs and pure Ni NWs. We believe that the above result suggests that the Fe(iii) ions incorporated both in the bulk and at the surface of the catalyst materials are responsible for the enhancement of the electrocatalytic performance.

##### (iv) Ease of charge transfer

Electrochemical impedance spectroscopy (EIS) analysis was performed at 1.55 V *vs.* RHE to further understand the possible origins of such superior electrochemical activity of NiFe (1 : 1) NWs. Fig. 4b shows that activated NiFe (1 : 1) possesses lower charge transfer resistance (*R*_CT_) ∼13 Ω than Ni (∼240 Ω), NiFe (2 : 1) (∼23 Ω) and NiFe (1 : 2) (∼135 Ω). Such high values of *R*_CT_ in Ni and NiFe (1 : 2), where Fe_3_O_4_ forms as a separate phase, suggest that the charge transfer ability of standalone nickel and iron centers is very low. In contrast, significant improvement of charge transfer ability occurs in materials where iron and nickel centers are in close proximity. We believe that the facile charge transfer ability of NiFe (1 : 1) is one of the defining factors behind its superior OER electrocatalytic activity.

#### Faradaic efficiency calculation and stability

The faradaic efficiency of the OER was evaluated by comparing the amount of experimentally evolved and theoretically calculated O_2_ (details in the ESI†). The faradaic efficiency for Ni NWs and NiFe (1 : 1) NWs was found to be 61% and 93.5%, respectively (Fig. 4c). This indicates that iron incorporation in nickel not only improves the electrocatalytic activity but also improves the four electron transfer process leading to higher production of oxygen. The performance of NiFe (1 : 1) under continuous usage was evaluated by 24 h of chronoamperometry at 1.56 V *vs.* RHE and ∼20 percent decrease in activity was observed over time (shown in Fig. S10†). The underlying reason behind the decrease of activity over time was later revealed from post-catalytic analysis (*vide infra*).

#### Post-catalytic analysis – the advantage of the “conductive core-active shell”

**Fig. 5:**
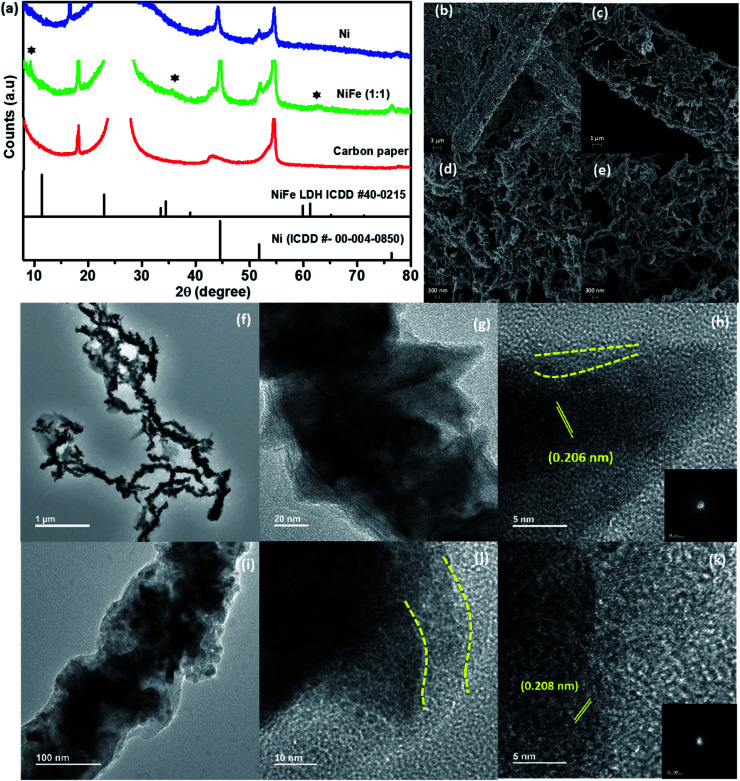
(a) XRD patterns of NiFe (1 : 1) and Ni after OER electrolysis, (b–e) SEM images of NiFe (1 : 1) after OER electrolysis, and (f–k) TEM and HRTEM images of NiFe (1 : 1) after OER electrolysis.

In recent times, it has been widely reported that after subjecting to electrocatalysis, materials undergo certain changes and the new species that gets generated act as the electrocatalytically active center. To understand the nature of the catalyst involved in the electrocatalytic process, both Ni and NiFe (1 : 1) were subjected to chronoamperometry treatment at high current densities of 50 mA cm_geo_^−2^ for 48 hours and subsequently analyzed through standard characterization techniques. In the grazing incidence X-ray diffraction patterns (GI-XRD patterns collected at an incident angle of 0.2°) of both post OER catalysts, peaks corresponding to the planes (111), (200), and (220) of Ni (ICDD #04-0850) and characteristic high intensity peaks at 18° and 26° from carbon paper were observed (shown in Fig. 5a). For NiFe (1 : 1), additional peaks at 2*θ* ∼ 10°, ∼35°, and ∼63° corresponding to the standard pattern of NiFe layered double hydroxides (LDHs) were observed. Literature reports suggest that this electrochemically generated NiFe LDH phase is highly potent catalytic centers to promote the OER.^43^ Interestingly, such additional peaks were absent in the PXRD pattern of post-catalytic Ni, thereby indicating that the formation of the catalytically active phases is less favourable in this case. It has been earlier reported that Fe^3+^ ions help in the stabilization of the LDH phases.^44^ We believe that the favourable generation of catalytically potent LDH phases in NiFe (1 : 1) over Ni may be responsible for the higher OER electrocatalytic activity of the former. The XPS analysis of post-OER materials (subjected to chronoamperometry at 10 mA cm_geo_^−2^) provided additional insights regarding the active catalytic centers. For post-OER Ni and NiFe (1 : 1), peaks of Ni 2p_3/2_ appeared at 855.8 eV and 855.7 eV, respectively. Literature reports suggest that they belong to the nickel centers in NiOOH (Fig. S11†).^45^ The XPS spectrum (shown in Fig. S4†) of O 1s showed signals at 533.12 eV and 535.29 eV, which indicate the presence of OH^−^ species. In the XPS spectrum of post catalytic materials, the complete disappearance of peaks from Ni(0) suggests that most of it at the surface has converted to the hydroxides/oxyhydroxides. Both SEM and TEM images shown in Fig. 5b–k reveal that the material largely retained its wire-like morphology and was uniformly dispersed on the carbon paper. Conspicuously, the HR-TEM analysis of post-OER catalysts reveals the formation of a thin amorphous layer on the material surface. In fact, this newly formed amorphous phase was found to be more prominent in NiFe (1 : 1) compared to only Ni. According to literature reports, the electrochemically generated metal oxyhydroxide phases are generally amorphous in nature.^46,47^ Thus, the microscopy data perfectly complement our earlier observation from PXRD on the generation of catalytically active hydroxide/oxyhydroxide phases in NiFe (1 : 1). Furthermore, the HRTEM images in Fig. 5h and k show that a phase with a lattice fringe of ∼0.210 nm (shown in Fig. 1d) is lying underneath the amorphous catalyst layer. This corresponds to the lattice plane (111) of nickel and therefore confirms that while the surface of the as-prepared nanowires transforms into the catalytically active sites, the interior retains the metallic character. Correlating the observations from GI-XRD, microscopy and XPS analyses for post-catalytic materials suggests that while the precondition by performing chronoamperometry for 60 seconds produces varying amounts of catalytically active nickel hydroxide on the surface of the materials, their interior remain as metallic nickel. Moreover, while the metal hydroxide formed at the surface further generates electrocatalytically active higher oxidation states of nickel during electrochemical treatment, the metallic nickel interior provides a highly efficient conductive support to the active catalyst centers generated on its surface. To confirm whether there is any change in the elemental ratio of nickel and iron, ICP-AES analysis was carried out and the results indicate that there is a slight increase in the nickel : iron ratio in the materials, in particular for NiFe (1 : 1), after electrocatalysis. Recently, Berlinguette *et al.* have also observed such an iron leaching phenomenon for nickel–iron based electrocatalysts.^48^ We believe that during the electrochemical surface reconstruction process, the iron species preferentially present at the surface of the materials cannot entirely get incorporated in the newly formed material and therefore leaches away during continuous usage. As already discussed, the incorporated iron ions are playing a promotional role in increasing the activity of the as-synthesized materials and as a result, any decrease in its concentration must lead to a decrease in activity. This was found to be true from the chronoamperometry study with NiFe (1 : 1), the activity of which decreased ∼20 percent over time as previously discussed.

## Conclusion

In this article, a simple one-pot method was employed to synthesize nickel nanowires and iron [Fe(iii)] incorporated nickel nanowires without the assistance of any templates and external magnetic field. Sharp nano-spikes were found to uniformly form on the surface of these nanowires. Detailed growth mechanism analysis reveals that the formation proceeds through a non-classical pathway of nanoparticle attachment. Furthermore, an “amorphous to crystalline” transformation of the as-synthesized materials was observed with increase in reaction time. The widely used precondition step of performing CV in the OER potential range was found to be not capable of generating electrocatalytically active sites. The precondition step was modified by prudent choice of potentials and electrochemical techniques and this resulted in significant improvement of the electrocatalytic OER activity. Rigorous post-catalytic analysis revealed that a very thin layer of amorphous nickel hydroxide gets generated on the surface of nanowires, particularly in the presence of iron(iii). While this layer at the surface efficiently electrocatalyzes the OER, the metallic nickel core provides the much-needed electrical conductivity. The iron incorporated nickel hydroxide was found to show significantly better activity from both “intrinsic” and “geometric” perspectives compared to only nickel nanowires. In contrast to earlier reports, Fe(iii) ions incorporated at both the surface and the bulk of nickel nanowires were found to participate in the promotion of the electrocatalytic OER activity.

This study offers a deep insight into the non-classical growth mechanism consisting of an “amorphous to crystalline” transformation for the synthesis of metallic nanowires. A suitable precondition strategy was devised to activate only the surface of the metallic nanowires that are otherwise inactive for the OER. The highly conductive nickel core and iron ions incorporated in both bulk and surface of the nanowires were found to promote the OER activity of the materials. We believe that this work will encourage the adoption of appropriate electrochemical treatments to activate the otherwise inert materials for OER electrocatalysis.

## Conflicts of interest

The authors declare no conflict of interest.

## Supplementary Material

NA-002-D0NA00073F-s001
