# Supplementary material for: Prudent electrochemical pretreatment to promote the OER by catalytically inert “Iron incorporated metallic Ni nanowires” synthesized via the “non-classical” growth mechanism
Source: Nanoscale Adv. 2020 Mar 16;2(5):1927–38. doi: 10.1039/d0na00073f (PMC9418993; doi:10.1039/d0na00073f)
Supplement: NA-002-D0NA00073F-s001 [file NA-002-D0NA00073F-s001.pdf]

Electronic Supplementary Material (ESI) for Nanoscale Advances.
This journal is © The Royal Society of Chemistry 2020

# Electronic Supporting Information

# Prudent electrochemical pretreatment to promote OER by catalytically inert "Iron incorporated metallic Ni nanowires" synthesized via "non–classical" growth mechanism

*Athma E. Praveen, Sagar Ganguli, and Venkataramanan Mahalingam**

Department of Chemical Sciences, Indian Institute of Science Education and Research (IISER)

Kolkata, Mohanpur, West Bengal, 741246, India.

Email: *mvenkataramanan@yahoo.com*

**INSTRUMENTATION**

The powder x-ray diffraction (PXRD) patterns were collected using the Rigaku-Smart Lab diffractometer attached with D/tex ultra detector and Cu $K_{\alpha}$ source operating at 35 mA and 70 kV. Scan range was set from 10-60° 2θ with a step size of 0.02° and a count time of 2 seconds. The samples were well powdered and spread evenly on a quartz slide. BET surface area measurements were carried out by $N_2$ adsorption at 77.3 K using a Quantachrome Novawin2 instrument. TEM measurement was carried out using a high resolution FEG transmission electron microscope (JEOL, JEM 2100F) with a 200 keV electron source. Briefly, a drop of the sample dispersed in water was drop casted on a strong carbon coated 300 mesh Cu grid and dried in air. Field emission scanning electron microscopy (FESEM) images were taken on the SUPRA 55-VP instrument with patented GEMINI column technology. Prior to the loading of the samples into the chamber, they were coated with a thin film of gold-palladium in order to avoid charging effects. XPS measurement was carried out using X-ray Photoelectron Spectroscopy (XPS) was recorded in PHI 5000 Versa Prob II. ICP AES analysis was carried out using ICP spectrometer (ARCOS, Simultaneous ICP Spectrometer).

**MATERIALS**

Nickel chloride ($NiCl_2$), ethylene glycol, Hydrazine monohydrate, and potassium hydroxide (KOH) and N-Methyl-2-pyrrolidone (NMP) were purchased from Merck. Ruthenium (IV) oxide anhydrous ($RuO_2$) and Polyvinylidene fluoride (PVDF) purchased from alfa aeser. Ferric chloride ($FeCl_3$) was procured from Sigma Aldrich. All chemicals were used without further purification.

**ELECTROCHEMICAL MEASUREMENTS**

Oxygen evolution reactions (OER) were performed in a three electrode glass cell on a CHI604D and Biologic SP300 electrochemical stations with an Ag/AgCl (3.5 M KCl) as the reference electrode and platinum wire as a counter electrode and the fabricated carbon paper based materials as the working electrodes in 1.0 M KOH solution. All potentials measured were calibrated to a reversible hydrogen electrode (RHE) using the following equation: $E_{RHE} = E_{Ag/AgCl} + 0.059*pH + 0.22$ V. Cyclic voltammograms were recorded at a scan rate of 5 $mVs^{-1}$ to obtain the polarization curves. All polarization curves are IR (75 %) and capacitance corrected.

**ELECTRODE PREPARATION FOR ELECTROCHEMICAL MEASUREMENT**

To prepare NiFe electrode, 3 mg sample was dispersed in 570 µl of ethanol and 30 µl PVDF solution (8 mg PVDF in 1 ml NMP). The mixture was then sonicated to get homogenous ink. 20 µl of the ink was dropcasted onto to the surface of 0.25 $cm^2$ carbon paper and dried. The amount of sample loaded onto the carbon paper was 0.4 $mg/cm^2$.

**KOH ELECTROLYTE PURIFICATION**

$Ni(NO_3)_2·6H_2O$ (2g) were dissolved in 4 mL of $H_2O$ and 20 mL of 1 M KOH to get $Ni(OH)_2$ precipitate. The mixture was shaken and centrifuged, and the supernatant was decanted. Resultant $Ni(OH)_2$ was washed with water and KOH for 3 times. Further, a 50 mL of 1M KOH was added to this washed $Ni(OH)_2$ for purification. The solution was stirred for 10 min, followed by at least 3 h of resting. The mixture was centrifuged, and the purified KOH supernatant was collected

**FARADAIC EFFICIENCY**

In order to quantify the Faradaic efficiency, the volume of the gas evolved was monitored by the displacement of the KOH electrolyte via inverted tube method. Chronoamperometry was performed for 20 min at 1.6 V vs RHE to quantify the charge and corresponding number of moles that should theoretically form was calculated using ideal gas law ($nO_2$ = PV/RT). Subsequently, the Faradaic efficiency was calculated using the expression.

$$\text{FE}(\%) = \frac{\text{Volume change observed}}{\text{Volume change theorectical}} * 100$$

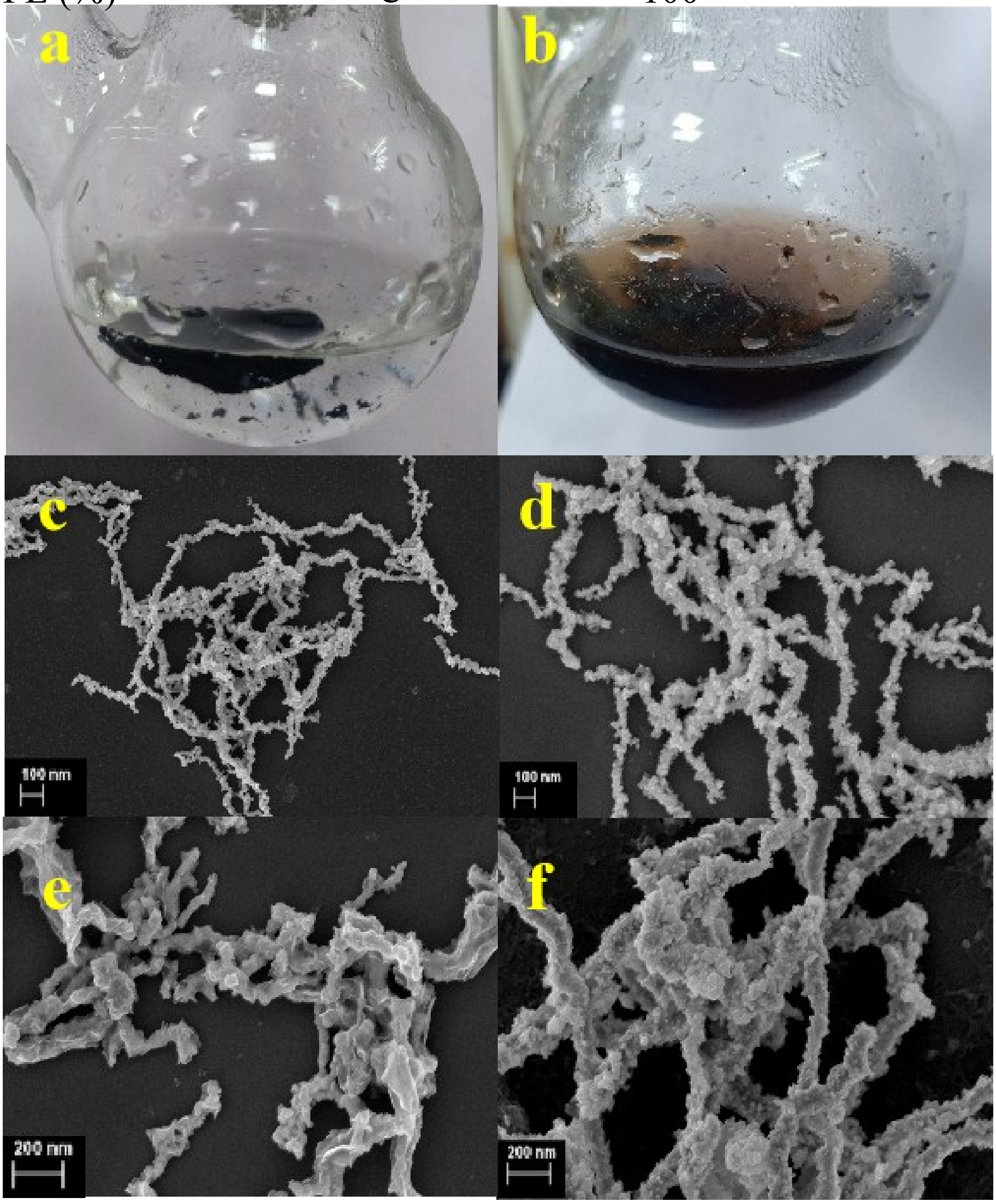

**Figure S1** Optical image as-synthesized (a) Ni NWs and (b) NiFe NWs. SEM images of (c) Ni, (d) NiFe(1:1), (e) NiFe(2:1), (f) NiFe(1:2).

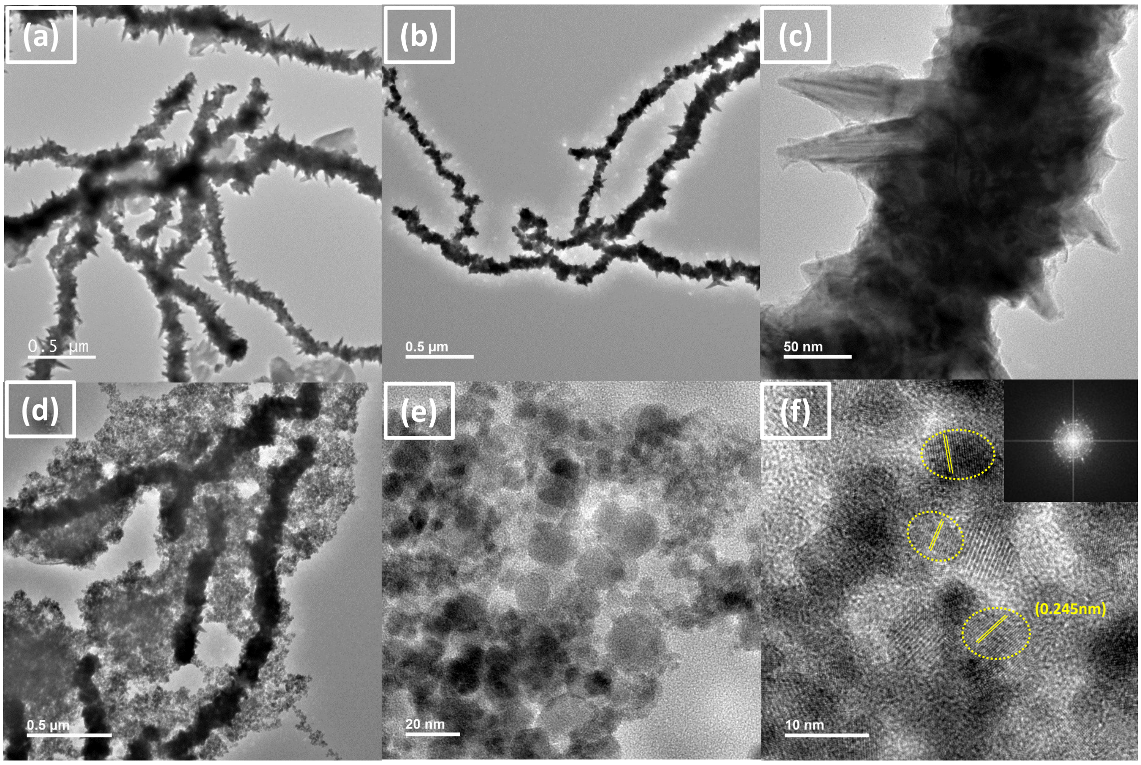


**Figure S2**. TEM images of (a-c) NiFe(2:1) and (d-f) NiFe(1:2) NWs.

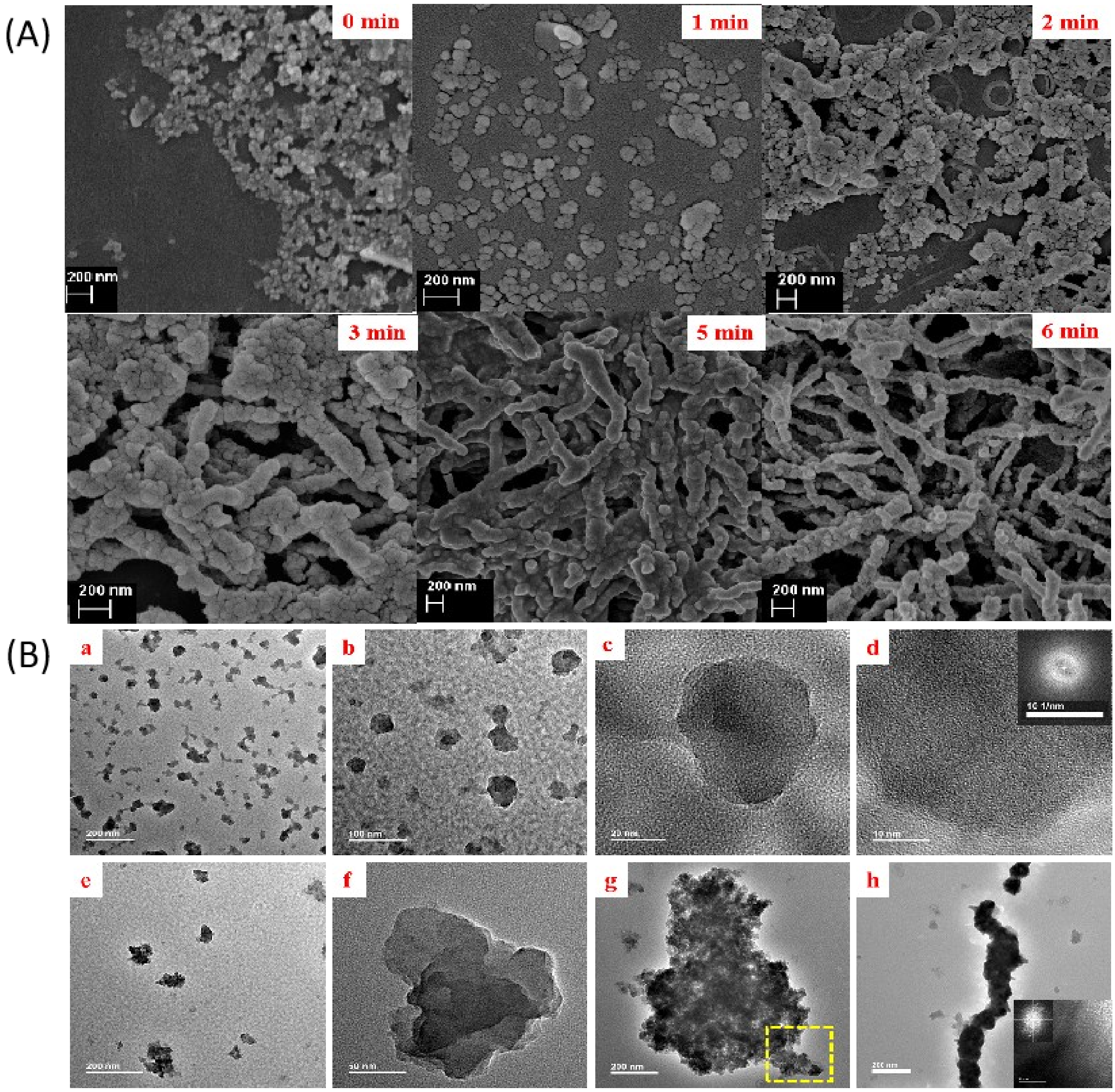


**Figure S3.** A) SEM images of NiFe NWs aliquots taken during the synthesis at different time. B) TEM images of NiFe NWs aliquots at ~0 sec (a-d) and 60 sec (e-f) after addition of $FeCl_3$.

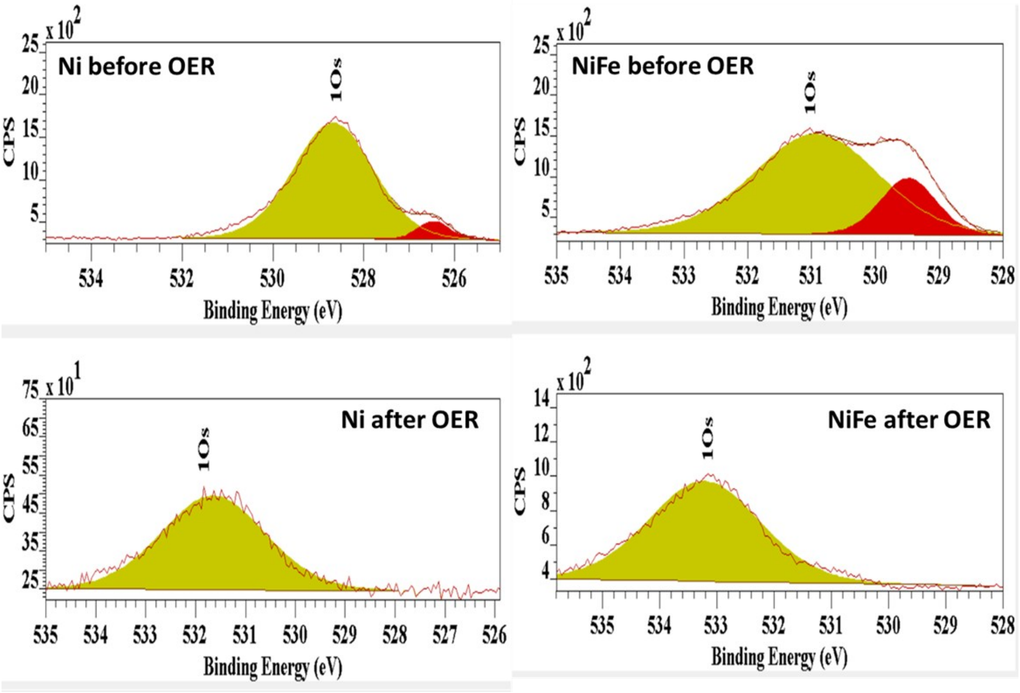


**Figure S4.** XPS spectra of O1s before and after catalysis.

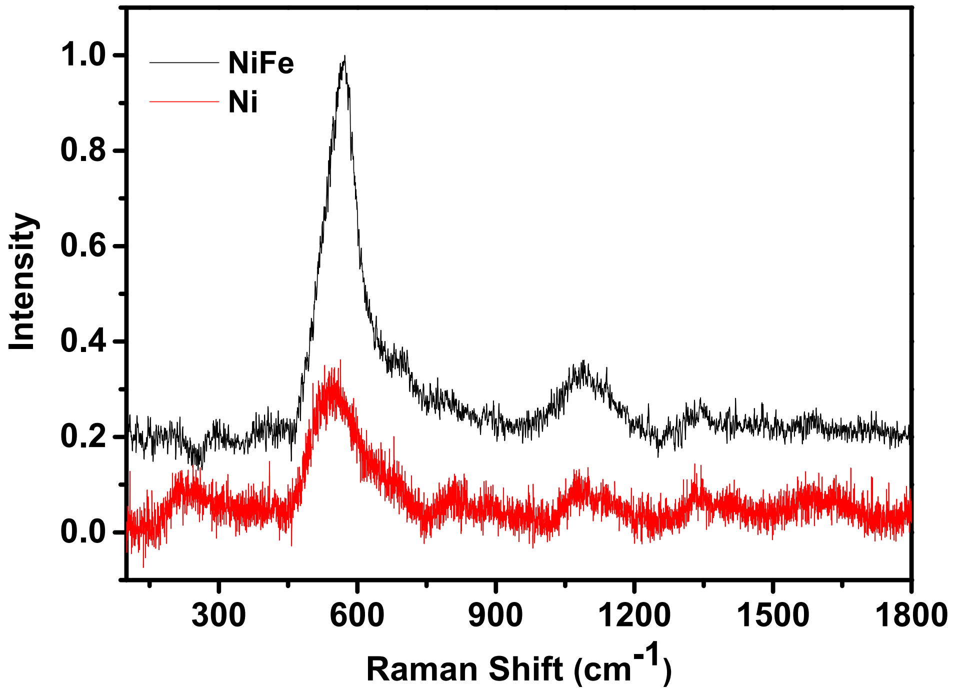


**Figure S5.** Raman spectra of Ni and NiFe NWs.

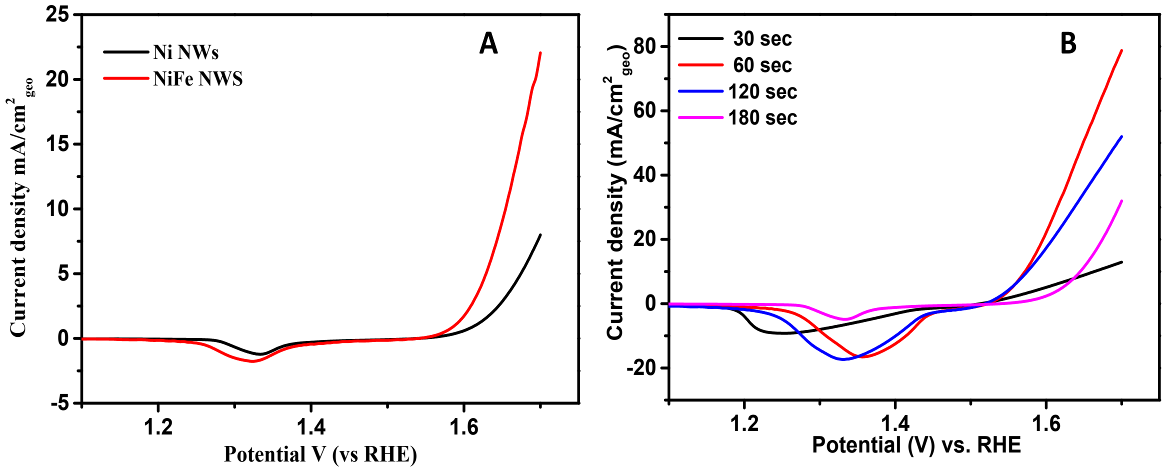


**Figure S6.** A) CV (backward scan) of NWs before the chronoamperometry. B) CV (backward scan) of NWs after the chronoamperometry at different time.

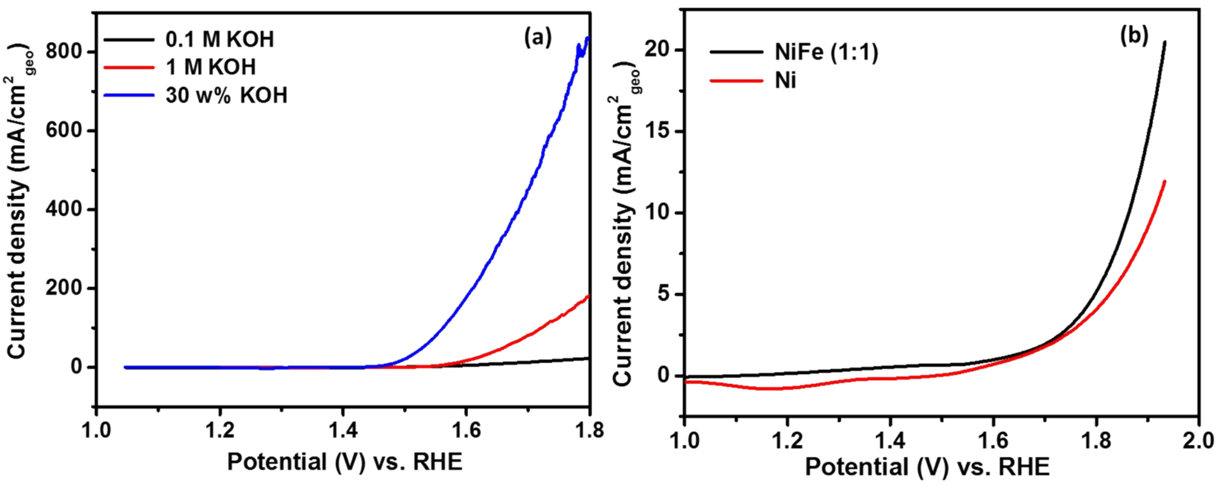


**Figure S7.** (a) CV curves for NiFe (1:1) NWs in 0.1M, 1M and 30 w% KOH, normalized by geometric surface area of electrodes (b) CV curves for Ni and NiFe (1:1), NWs in 1M PBS, normalized by geometric surface area of electrodes.

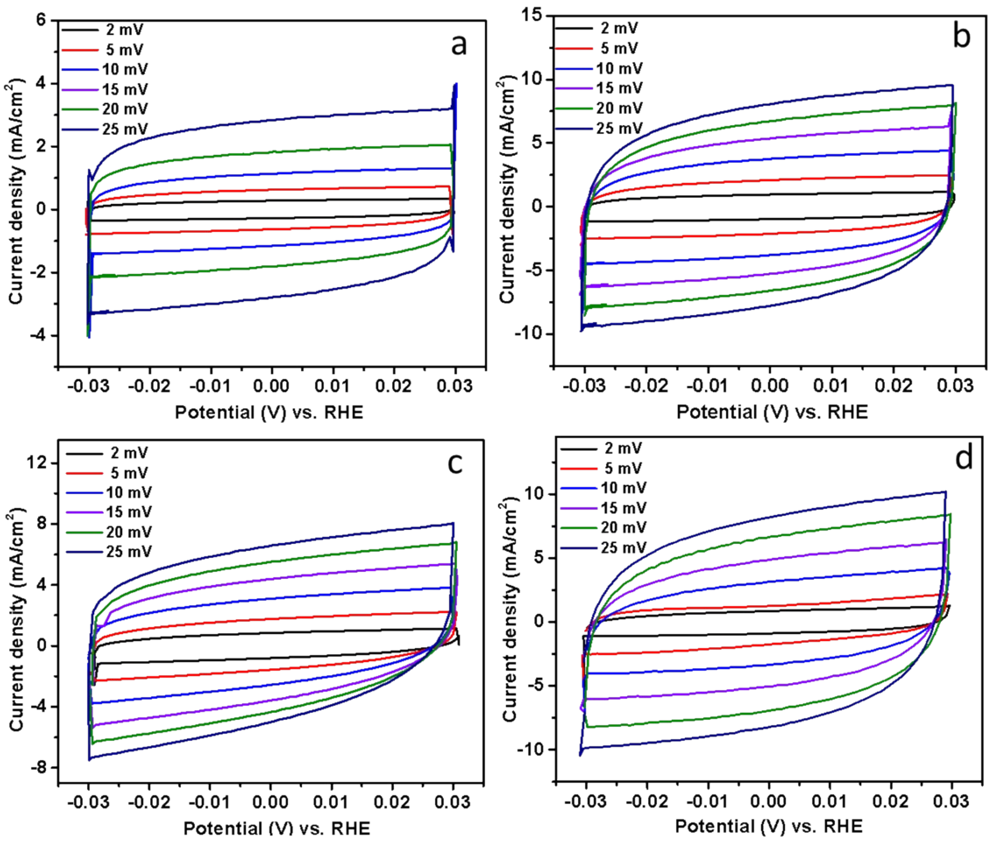


**Figure S8.** Cyclic voltammetry curves of (a) NiFe (1:1), (b) Ni, (c) NiFe (2:1) and (d) NiFe (2:1) at different scan rates.

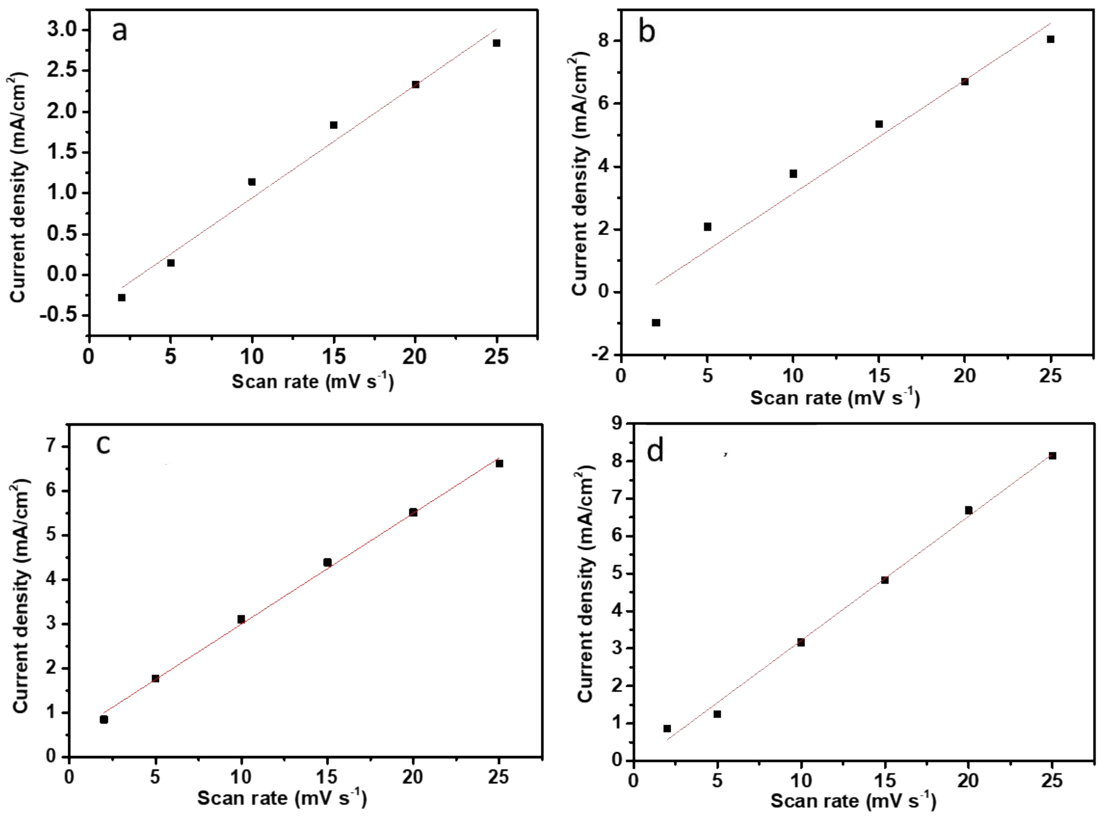


**Figure S9.** Scan rate dependence of the current densities of a) NiFe (1:1), b) Ni, c) NiFe (1:2) and d) NiFe (2:1) NWs.

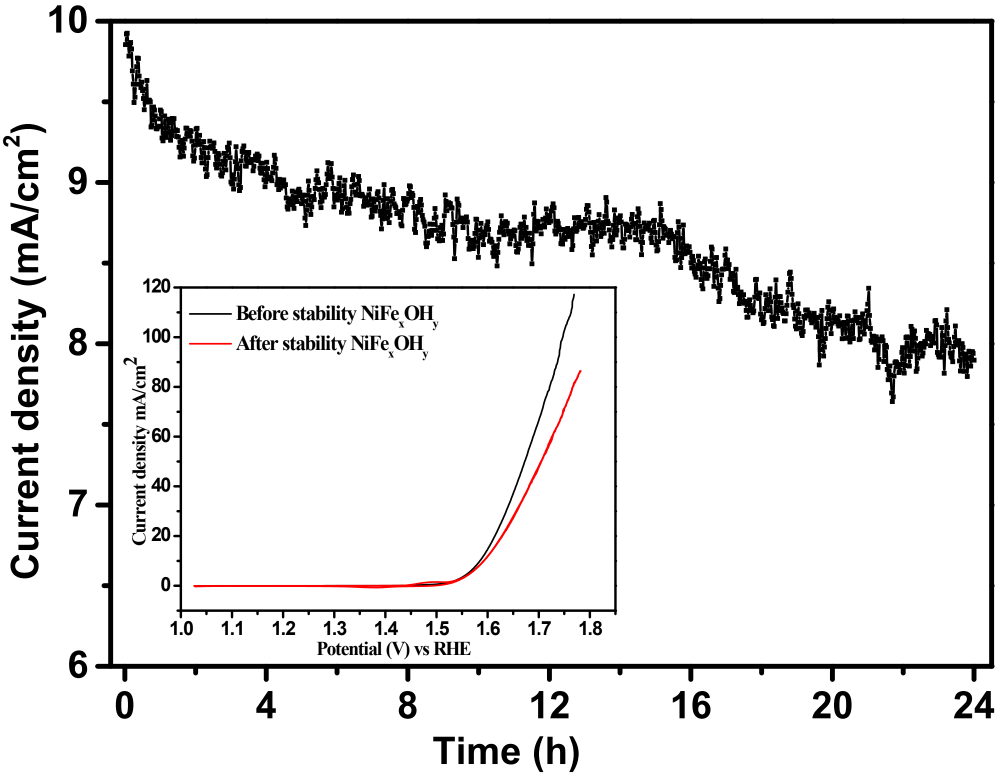


**Figure S10.** Chronoamperometry of NiFe (1:1) NWs for 24h at 1.54 V.

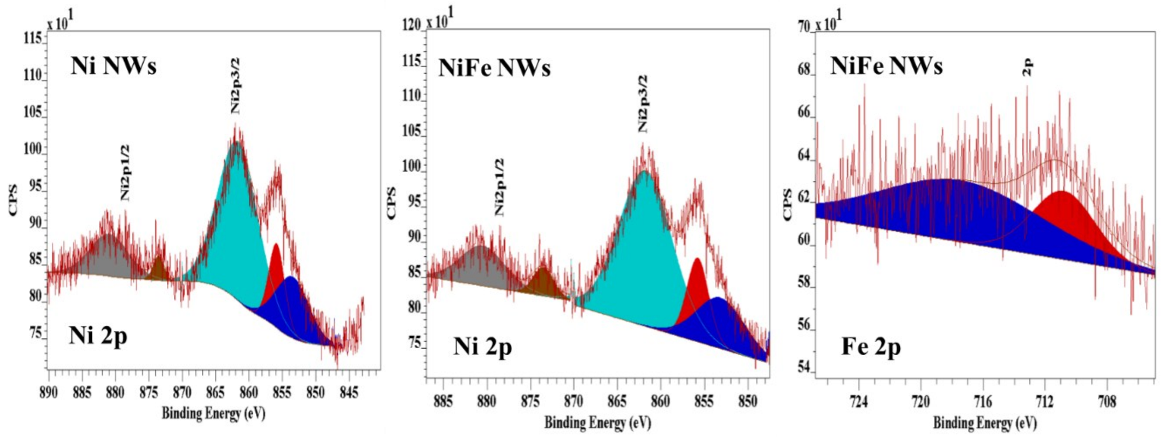


**Figure S11.** XPS spectra of post OER Ni, NiFe (1:1) samples.

**Table S1.** Comparison of NiFe nanowire on carbon paper with reported active electrocatalysts towards OER under alkaline conditions.

| No | Material | OER overpotential (mV) for 10mA/cm$^2$ (only iR corrected) | OER overpotential (mV) for10mA/cm$^2$ (capacitance, redox current and iR corrected) | Reference |
|---|---|---|---|---|
| 1 | **NiFe(OH)x /CP** | 500 | | J. Phys. Chem. C, 2015, 119, 19573. |
| 2 | **$Ni_2P$/FTP nanowire** | 420 | | Chem. Commun., 2015, 51, 11626. |
| 3 | **CoP@NiFe–OH/SPNF** | 300 | | Nanoenergy, 2019, 10382. |
| 4 | **$NiFe_xOH_y$@Ni NW/ CP** | | **330** | **This work** |
| 5 | **NiFe(OH)x/FeS/IF** | 270 | | Adv. Funct. Mater. 2019, 1902180. |
| 6 | **(Ni-Fe)$S_x$/NiFe$(OH)_y$ /NF** | 290 | | Applied Catalysis B: 246,2019, 348. |
| 7 | **NiFeV LDHs /NF** | 200 | | Adv. Energy Mater. 2018, 8, 1703341. |
| 8 | **$(Pr_{0.5}Ba_{0.5})CoO_{3-x}$ (0.1M KOH)** | | 310 | Nat. Commun. 2013, 4, 2439. |
| 9 | **$(Ba_{0.5}Sr_{0.5}Co_{0.8}$ $Fe_{0.2}O_{3-x}$/AC black (0.1M KOH)** | | 320 | Adv. Energy Mater. 2015, 5, 1402033. |
| 10 | **TiN@$Ni_3N$ nanowire** | 350 | | J. Mater. Chem. A, 2016, 4, 5713 |

**Table S2.** ICP AES analysis of NiFe NWs before and after electrocatalytic analysis

| Sample (mM) | Ni(ppm)/ | Fe (ppm) | Ni:Fe ratio |
|---|---|---|---|
| **NiFe (1:1)** | 63.522 | 4.839 | 11.62 :1 |
| **NiFe (1:2)** | 25.959 | 24.343 | 1.01 :1 |
| **NiFe (2:1)** | 50.083 | 0.106 | 473.8 :1 |
| **NiFe (1:1) After catalysis** | 68.333 | 4.176 | 15.56 :1 |
